# Halogen Bonding and Rearrangements in Complexes of *N*-Chlorosuccinimide with Halides

**DOI:** 10.3390/molecules30030639

**Published:** 2025-01-31

**Authors:** Maison Hardin, Matthias Zeller, Sergiy V. Rosokha

**Affiliations:** 1 Department of Chemistry, Ball State University, Muncie, IN 47306, USA; maison.hardin@bsu.edu; 2 Department of Chemistry, Purdue University, West Lafayette, IN 47907, USA; zeller4@purdue.edu

**Keywords:** halogen bonding, *N*-chlorosuccinimide, halides, X-ray crystallography, DFT calculations

## Abstract

The role of halogen bonding (HaB) in the reactions of *N*-chlorosuccinimide (SimCl), a versatile reagent in organic synthesis, was investigated through experimental and computational analyses of its interactions with halides. The reactions of SimCl with Br^−^ or I^−^ resulted in the crystallization of HaB complexes of chloride with *N*-iodosuccinimide (SimI) or *N*-bromosuccinimide (SimBr). Computational analysis revealed that halogen rearrangements, which occurred even at −73 °C, were facilitated by halogen bonding. The dissociation of SimCl∙Y^−^ (Y = I or Br) complexes into a Sim^−^ + ClY pair (followed by the rotation and re-binding of the interhalogen molecules) bypassed the formation of the high-energy Sim^−^ + Cl^+^ pair and drastically (about tenfold) reduced the dissociation energy of the N–Cl bond. Furthermore, while the dissociation energy of individual SimCl is higher (and its HaB is weaker) compared to that of SimI or SimBr, the dissociation of the N-Cl bond in SimCl∙Y^−^ requires less energy than in the complexes of SimBr or SimI. The facile cleavage of such bonds in HaB complexes explains the high reactivity of SimCl and its effectiveness as a halogenating agent.

## 1. Introduction

Halogen bonding, an attraction between electron-deficient halogen atoms and an electron-rich species, has emerged in recent years as a powerful tool in crystal engineering and molecular recognition [1,2,3,4]. Numerous studies have also demonstrated its substantial role in the reactions of halogenated molecules and its utility in catalysis [5,6,7,8]. In particular, both experimental and computational analyses have shown that strong halogen bonding can facilitate electron and/or halogen transfer between interacting species [9,10,11,12]. The latter can be viewed as a subclass of nucleophilic substitution reactions, referred to as halophilic or S_N_2X substitutions, in which the nucleophile attacks a halogen substituent instead of the carbon atom in a C–X bond [13].

It should be noted, however, that most studies of halogen bonding have focused on iodo- and bromo-substituted molecules [1,2]. This choice is related to the better polarizability of these heavier halogen atoms, which results in much stronger halogen bonding. Halogen bonding via chlorine in most cases is relatively weak [14,15,16,17], and the role of this interaction in the reactions of chloro-substituted molecules remains largely unexplored.

**Figure 1:**
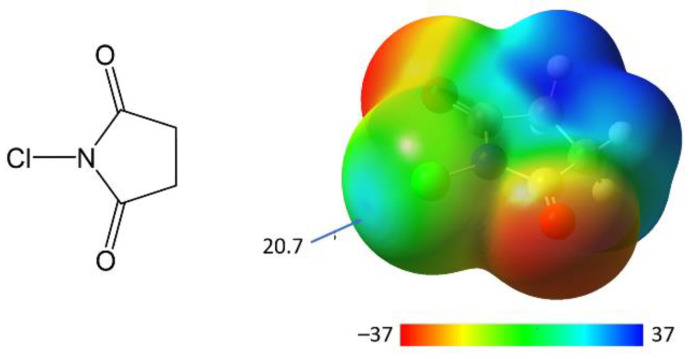
Structure and surface electrostatic potential of SimCl (in kcal/mol at 0.001 a.u. electron density).

Given this context, it is of interest to evaluate the role of halogen bonding in the reactions of *N*-chlorosuccinimides (SimCl). This molecule is a representative of *N*-haloimides, common halogenating agents in which halogens are bonded to nitrogen atoms [18,19,20]. Furthermore, *N*-iodosuccinimide (SimI) and *N*-bromosuccinimide (SimBr) are known to be very strong halogen-bond (HaB) donors, forming exceptionally short halogen bonds with a variety of nucleophiles [21,22,23,24]. In contrast, despite the presence of a σ-hole on the surface of the chlorine atom in *N*-chlorosuccinimide (SimCl, Figure 1) [25,26], which is comparable in strength (~21 kcal/mol at 0.001 a.u. electron density) to that of the surfaces of iodine and bromine in well-known HaB donors (e.g., bromoform [27]), attempts to obtain HaB complexes of SimCl have been mostly unsuccessful.

For example, Stilinović et al. reported X-ray structures of several HaB complexes of SimI and SimBr with various pyridines but noted that the isolation and characterization of similar complexes with SimCl was hindered by rapid decomposition [25]. Our recent study showed that the interaction of the structurally similar *N*-chlorosaccharin with quinuclidine led to chlorine transfer, yielding HaB complexes between *N*-chloroquinuclidinium cations and chloride instead of a halogen bonded complex of chlorosaccharin [26]. As such, the 2:1 complex of SimCl with chloride remains the only halogen-bonded association of this molecule reported in the Cambridge Crystallographic Data Centre [28]. In comparison, earlier studies on the interaction of SimCl with bromide resulted in the formation of *N*-bromosuccinimide, while reactions of SimI with iodide produced *N*-iodosuccinimide [29,30]. Eberson et al. hypothesized that such halogen exchanges might occur through a halophilic process involving an activated complex with partial bonds between chlorine and bromide or via an intracomplex electron-transfer mechanism [29]. However, the absence of experimental or computational evidence has made it difficult to validate these pathways.

The wide-ranging applications of *N*-chlorosuccinimide as a versatile reagent in organic synthesis [31,32,33] underscore the importance of clarifying the role, if any, of halogen bonding in its reactions. Moreover, this electrophilic molecule seems ideally suited for exploring the potential effects of halogen bonding in the reactions of chlorinated molecules in general. Therefore, in the current work, we conducted experimental and computational investigations into the interactions of SimCl with the simplest monoatomic nucleophiles—bromide and iodide anions.

## 2. Results and Discussion

### 2.1. X-Ray Structural Characterization of the Products of Interaction of SimCl with Bromide and Iodide

**Figure 2:**
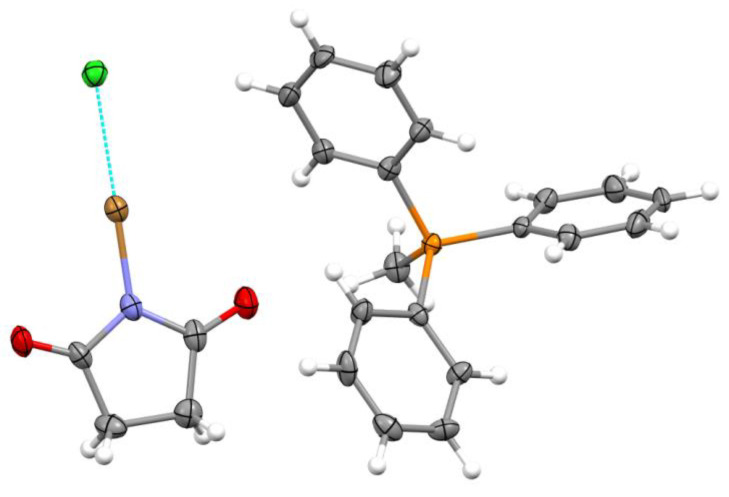
X-ray structure of **1** resulting from the interaction of SimCl with [Me(Ph_3_)P]Br. Color code: dark gray—carbon, light gray—hydrogen, blue—nitrogen, red—oxygen, brown—bromine, orange—phosphorous and green—chlorine.

The addition of methyltriphenylphosphonium bromide to a solution of SimCl in dichloromethane and the slow evaporation of the resulting mixture at room temperature (see Experimental section) produced colorless crystals **1**. The FT-IR spectrum of these crystals showed a substantial red shift in the C=O vibration frequencies and a blue shift in the C-N stretching frequencies as compared to those of SimCl (Figure S1 and Table S1 in the Supporting Information). X-ray crystallographic analysis revealed that the succinimide moieties in these monoclinic crystals (*P*2_1_ space group, see Table S2 in the Supporting Information for crystallographic, data collection, and refinement details) are bonded to bromine atoms, which are, in turn, halogen-bonded to chloride anions (Figure 2). In other words, the interaction of SimCl with bromide resulted in halogen exchange and the formation of 1:1 HaB complexes between the resulting SimBr and chloride.

The SimBr·Cl^−^ complexes are nested between the benzene rings of the bulky counter-ions (see crystal packing in Figure S2 in the Supporting Information). The Br⋯Cl distance of 2.659 Å (Table 1) in these complexes is 26% shorter than the sum of the van der Waals radii of bromine and chlorine atoms [34], indicating a very strong interaction between these species. The N–Br bond length of 1.919 Å in SimBr is elongated compared to the analogous bond in individual SimBr (1.836 Å [35]) and in the previously reported 2:1 complex of SimBr with chloride (~1.88 Å [36]).

**Figure 3:**
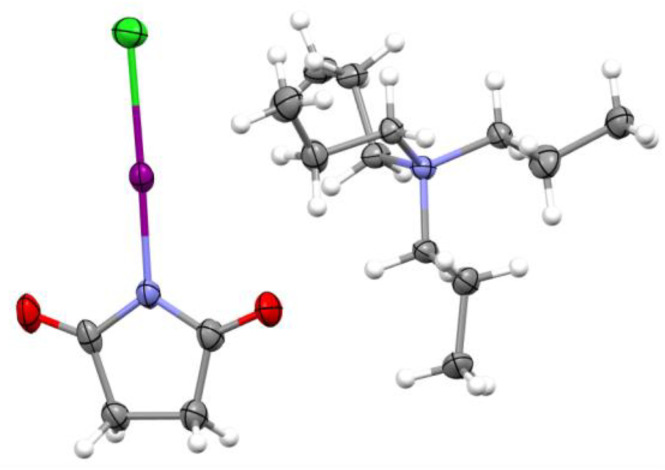
X-ray structure of **2** resulting from the interaction of SimCl with [Pr_4_N]Br. Color code: dark gray—carbon, light gray—hydrogen, blue—nitrogen, red—oxygen, magenta—iodine and green—chlorine.

A similar evaporation of a dichloromethane solution containing a mixture of SimCl and iodide (taken as a salt with *n*-tetrapropylammonium as the counterion) produced crystals **2** (see crystal packing in Figure S3 in the Supporting Information). The changes in the IR spectra of these crystals compared to SimCl were similar (e.g., a red shift in the C=O stretching vibrations around 1700 cm^−1^ and a blue shift in the C-N stretching vibrations in 1150–1200 cm^−1^ range) as those in the crystals **1** (Figure S1 in the Supporting Information). These colorless monoclinic plates **2** also comprised the product of halogen exchange, i.e., the SimI·Cl complex in which the succinimide moiety is bonded to iodine atoms, which are, in turn, bonded to chlorine (Figure 3).

The I⋯Cl distance in the SimI·Cl^−^ complexes is 29% shorter than the sum of the van der Waals radii of chlorine and iodine and the Br⋯Cl distance in the complexes of Cl^−^ with SimBr in crystals **1**. Also, the N–I bond in the SimI·Cl complexes (Table 1) is significantly elongated compared to the bond in individual SimI (2.06 Å [37]). As with the Br⋯Cl distance, the I⋯Cl bond in the SimI·Cl complexes is shorter and the N–I bond is longer than that measured in the similar 2:1 complexes [36] and in the associations in which Cl^−^ is halogen bonded both with SimI and 1,3-bis(2,6-diisopropylphenyl)-2-iodo-imidazolium [38].

To determine whether lowering temperatures (which commonly slow down reactions) can suppress the halogen exchange, we conducted crystallization of the HaB complexes by diffusing hexane into dichloromethane solutions of SimCl with bromide or iodide salts at −70 °C to −75 °C. In the systems containing SimCl together with methyltriphenylphosphonium iodide, such low-temperature crystallizations produced colorless plates (crystals **3**). Similarly to the room-temperature crystallization, these monoclinic crystals (space group *P2_1_*) comprised the product of halogen exchange, i.e., a 1:1 HaB complex between SimI and the chloride anion (Figure S4 in the Supporting Information). Although the counter-ions in crystals **2** and **3** were different (Pr_4_N^+^ vs. MePh_3_P^+^), the geometric characteristics of the SimI·Cl complexes in these crystals were very similar (Table 1).

The analogous low-temperature diffusion of hexane into a solution of SimCl and methyltriphenylphosphonium bromide produced a mixture of colorless prisms (crystals **4**) and plates (crystals **5**). X-ray structural analysis revealed that both types of crystals contained HaB complexes, with chlorine and bromine partially disordered between two sites (Figures S5 and S6 in the Supporting Information). Specifically, one bromine atom was covalently bonded to the nitrogen atom, while the other occupied the terminal halogen position in the complex. The same was applied to chlorine. The product of halogen exchange (where bromine is covalently bonded to nitrogen) represented the predominant form in both crystals **4** and **5**, but the disorder ratios differed between the two. Specifically, the disorder ratio was 0.822(2):0.178(2) in the orthorhombic prisms **4**, whereas the ratio was 0.940(3):0.060(3) in the monoclinic plates **5**. Due to the small fraction of disordered bromine at the terminal positions, the unit cell geometry of the latter was essentially identical to that of the related crystals **1** (see Table S2 in the Supporting Information).

Overall, the results of the low-temperature crystallization demonstrated that addition of iodide or bromide to the solution of SimCl results in halogen exchange even at −70 °C to −75 °C. Under these conditions, the chlorine-to-iodine exchange proceeds much faster than crystallization, so the resulting crystals did not contain the original SimCl. However, the rate of chlorine-to-bromine exchange is apparently comparable to the crystallization kinetics. As a result, the resulting crystals contained minor fractions of the starting SimCl molecules. To clarify the mechanisms underlying these processes and the reasons for the differences observed in the systems with bromide and iodide anions, we conducted a computational analysis of the bonding in these systems.

### 2.2. Computational Analysis of the Bonding in the Complexes of N-Chlorosuccinimides with Halides

The M06-2X/def2-tzvpp computations (see the Materials and Methods for details) produced HaB complexes between SimBr or SimI molecules and Cl^−^ anions similar to that measured in the solid state. In particular, the optimized complexes showed Br⋯Cl and I⋯Cl separations, which were 23 and 29% shorter, respectively, than the van der Waals separations (Table 2). The N-Br and N-I bonds were elongated as compared to those in individual SimI and SimBr (listed in Table S3 in the Supporting Information). These characteristics indicate strong halogen bonding in these complexes. In comparison, the Cl⋯I and Cl⋯Br separations in the optimized SimCl·I^−^ and SimCl·Br^−^ complexes were about 10% shorter than the sums of the van der Waals separations, and the N-Cl bond was within 0.03 Å of that of SimCl. Following the distinction in the HaB bond lengths, the bindings energies of the terminal halogens in the SimI·Cl^−^ and SimBr·Cl^−^ complexes were substantially larger than in the similar complexes formed by SimCl with various halides.

In agreement with the experimental data, the optimized SimI·Cl^−^ and SimBr·Cl^−^ complexes showed substantial shifts in the C=O and C-N vibrations as compared to those in the individual SimCl molecule (Figure S1 in the SI). The corresponding shifts in the SimCl·I^−^ and SimCl·Br^−^ complexes were much smaller. Such distinctions are related to the differences in the charge transfer from the halides to the succinimide moieties in these complexes (which weaken C=O and strengthen C-N bonds). Specifically, the Natural Bond Orbital (NBO) analysis [39] revealed that the negative charges are predominantly localized on the terminal halogen atoms in all these complexes, while the central halogens exhibit partial positive charges. However, the terminal chlorine atoms in the SimI∙Cl^−^ and SimBr∙Cl^−^ complexes carry noticeably less than a unit negative charge, which is characteristic of halide anions. Accordingly, substantial negative charge also resides on the succinimide moieties (approximately −0.6 *e* and −0.4 *e* in SimI∙Cl^−^ and SimBr∙Cl^−^, respectively). In contrast, the charges on the terminal halogens in the complexes formed by SimCl are within 5% of a unit negative charge, with only about −0.27 *e* residing on the succinimide moieties.

To further clarify the differences between the X⋯Cl halogen bonding involving chlorine atoms in the SimCl complexes and those that formed by SimI or SimBr with chloride anions, we performed a QTAIM analysis [40,41] of the optimized SimX∙Y^−^ associations. The electron densities at the bond critical points (BCPs) along the Cl⋯I and Cl⋯I bond paths in the SimCl∙I^−^ complexes obtained from this analysis (Table 3) are close to 0.01 a.u. The energy densities at these points are very small and positive. These characteristics are typical of moderately strong, electrostatically driven supramolecular interactions [41].

In contrast, the electron densities at the BCPs along the X⋯Cl bond paths in the SimX∙Cl^−^ complexes are significantly higher than 0.01 a.u., and the energy densities at these BCPs are negative. These values suggest a partially covalent nature of the halogen bonding in the SimI∙Cl^−^ and SimBr∙Cl^−^ complexes [41,42,43]. Consistent with the distinctions in electron and energy densities at the BCPs, the electron localization function (ELF) values along the X⋯Cl bond paths in the SimI∙Cl^−^ and SimBr∙Cl^−^ complexes are much higher than those in the SimCl∙Y^−^ complexes (Table 3).

The increase in the values of *ρ*(r) and ELF on the X⋯Y bond paths in the SimI∙Cl^−^ and SimBr∙Cl^−^ complexes, compared to the corresponding values in the SimCl complexes, is accompanied by a substantial decrease in these values on the N-X bond paths. This indicates that as the strength of halogen bonding increases, the adjacent covalent bond involving the halogen atom weakens (Note that the strength of the I-Cl bonding in the associations formed by SimI and chloride suggests that it be alternatively considered as a complex between interhalogen ClI and Sim^−^. Yet, since the data in Table 3 indicate that N-I interaction is somewhat stronger than I-Cl bonding, the SimI∙Cl^−^ seems preferable).

Most importantly, the energies of the optimized SimI∙Cl^−^ and SimBr∙Cl^−^ complexes are lower than those of the corresponding SimCl∙I^−^ and SimCl∙Br^−^ associations with the same composition. Specifically, the halogen rearrangement converting SimCl∙I^−^ into SimI∙Cl^−^ lowers the energy of the system by 15.4 kcal/mol and the free energy by 14.0 kcal/mol. The energy changes resulting from the rearrangement of SimCl∙Br^−^ into SimBr∙Cl^−^ are smaller, with energy decreasing by 2.1 kcal/mol and free energy by 1.5 kcal/mol. The halogen exchange occuring without involvement of the HaB Complexes (i.i., starting from the separate SimCl and I^−^ or Br^−^ and producing separate Cl^−^ and SimI or SimBr) is less termodynamically favorable. Specifically, *ΔG* = −2.1 kcal/mol in the case of such Cl/I exchange, and the free energy of the separate SimBr and Cl^−^, is higher by 2.8 kcal/mol than the energy of the intial SimCl and Br^−^.

These data indicate that halogen bonding resulting in the formation of the SimI∙Cl^−^ and SimBr∙Cl^−^ complexes makes the halogen rearrangement a thermodynamically favored process in both systems. However, besides thermodynamics, the possibility of rearrangement is controlled by the kinetics of these processes. Since the rearrangement involves breaking the N-Cl bonds, the reaction rates are determined by the energies required for their dissociation. It should be noted in this respect that the energy of heterolytic dissociation of SimCl into Sim^−^ and Cl^+^ is very large (214 kcal/mol, see Table S3 in the Supporting Information). The homolytic dissociation of this molecule is less energy-intensive, with an energy increase of 79 kcal/mol. Nevertheless, any process involving such dissociations would be too slow to account for the halogen rearrangements observed at room or at low temperatures. Another possible pathway starts with electron transfer from iodide or bromide, followed by the easy dissociation of the SimCl^−·^ anion radical. However, the energy of the pairs resulting from such dissociation (e.g., 88 kcal/mol relative to the starting reactants for SimCl^−·^+ I^·^ pair) are also too high.

Halogen bonding of SimCl with halides, however, dramatically reduces the dissociation energy of the N-Cl bond. For instance, the dissociation of the SimCl∙Br^−^ complex into a succinimide anion and a ClBr molecule is accompanied by an energy increase of 24.5 kcal/mol and an even smaller free-energy change (Table 4).

Furthermore, the dissociation of the SimCl∙I^−^ complex into a succinimide anion and a ClI molecule results in a free energy increase of only 7.4 kcal/mol. It should be also noted that while the negative charges in the SimCl∙I^−^ and SimCl∙Br^−^ complexes reside primarily on the terminal halogen atoms (*vide supra)*, the homolytic dissociations of these complexes into Sim^·^ radicals and ClY^−·^ anion-radicals require significantly higher energy than the dissociations into succinimide anions and neutral ClY molecules (Table 4).

**Figure 4:**
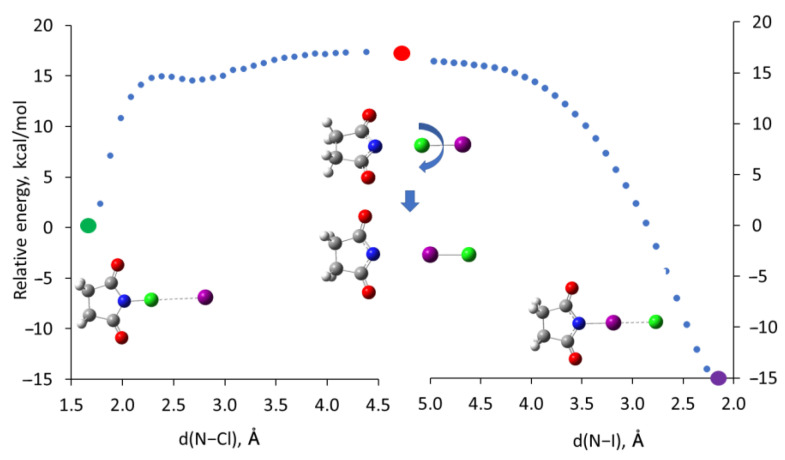
Energy scan along the N-Cl and N-I bonds in the SimCl∙I^−^ and SimCl∙I^−^ complexes (relative to the energy of SimCl∙I^−^). Green and purple circles represent the energies of the optimized SimCl∙I^−^ and SimCl∙I^−^ complexes, respectively, and the red circle shows the sum of the energies of the individual SimCl^−^ anion and ClI molecule.

The analysis of bonding indicates that the halogen rearrangements can be described as dissociation of the SimCl∙Y^−^ complexes into succinimide anions and ClY molecules, followed by the rotation and binding of ClY to Sim^−^ via iodine or bromine which produces more thermodynamically stable complexes. The energy scan (Figure 4, see Materials and Methods for details) along the N-Cl and N-I bonds indicates that the dissociations of the former and formation of the latter occur essentially without barriers.

Taking the energies of the pairs of dissociated Sim^−^ and ClY species as the barriers for the rearrangements allows for estimating the rates of these processes (see the Experimental section for details). In particular, the free energy of the Sim^−^ + ICl pair relative to the SimCl∙I^−^ complex of 7.4 kcal/mol implies that the rate constants of the rearrangement are on the order of 10^7^ s^−1^ at 298 K and 10^4^ s^−1^ at 200 K. This corresponds to a half-life of the SimCl∙I^−^ complex of less than a millisecond (even at −73 °C), which is much faster than the rate of crystallization. The estimated rate of the rearrangement of the SimCl∙Br^−^ complex at room temperature, about 10^2^ s^−1^ (with t_1_/_2_~0.01 s), is also faster than the crystallization kinetics. However, the estimated rate constant of this process at −73 °C (approximately 10^−4^ s^−1^) corresponds to a half-life of SimCl∙Br^−^ on the order of a few hours under these conditions. This timescale is comparable to the rates of crystallization and implies (in agreement with the experimental data) that some of the starting complex can co-crystallize with the product of the rearrangement.

The data in Table 4 also show that the dissociation energies (and the corresponding free energies) of the SimCl∙I^−^ and SimCl∙Br^−^ complexes are lower than those of the SimI∙Cl^−^ and SimBr∙Cl^−^ analogs. This is surprising since the heterolytic and homolytic dissociation energies of the individual SimCl molecule are higher than those of SimI and SimBr (Table S3 in the Supporting Information). Moreover, the halogen bonds of SimI or SimBr with chloride are stronger than those of SimCl with bromide or iodide which suggests a more substantial weakening of the N-Br and N-I bonds in the HaB complexes of SimI and SimBr. (This suggestion is consistent with the trend in N-X bond length increase and QTAIM characteristics in Table 2 and Table 3). However, the magnitude of the (negative) free energy change in the reaction of the Cl^+^ cation (formally released by SimCl) with I^−^ (or Br^−^) anions is much higher than that of the reaction of I^+^ (or Br^+^) with Cl^−^. This difference (approximately 77 kcal/mol for Cl^+^/I^−^ vs. I^+^/Cl^−^ reactions, see Table S4 in the Supporting Information for details) more than compensates for the difference in the N-X bond dissociation energies and explains the counter-intuitive trend in the thermodynamics of the processes with different *N*-halosuccinimides. Furthermore, halogen bonding eliminates the need to form the high-energy Sim^−^ + X^+^ pair resulting from the dissociation of the individual SimCl (since dissociation of the HaB complex proceeds directly to the Sim^−^ + XY pair without an additional energy barrier), which accounts for the fast kinetics of the rearrangement.

## 3. Conclusions

The crystallization of SimCl with Br^−^ and I^−^ anions results in halogen rearrangements and the formation of HaB complexes of SimI or SimBr with chloride. Computational analysis shows that these rearrangements (which proceed efficiently even at −73 °C) are facilitated by halogen bonding via chlorine atoms. Such SimCl∙Y^−^ complexes dissociate directly into the Sim^−^ + XY pair (followed by the rotation and re-binding of the interhalogen molecule via heavier atom which leads to the more stable complex). The involvement of HaB complexes makes halogen exchange thermodynamically favorable in the solutions of SimCl with I^−^ and Br^−^. It also eliminates the need for the formation of the high-energy Sim^−^ + X^+^ pair and dramatically (about tenfold) decreases the dissociation energy of the N-Cl bond.

Furthermore, the formation of the HaB complexes and their facile dissociation into succinimide anions and interhalogens represent, in essence, halophilic or S_N_2X substitution reactions (in which nucleophiles attack halogen substituents and replace the group originally bonded with this atom). Thus, our results show that even relatively weak halogen bonding via chlorine atoms plays a critical role in these processes.

Notably, the dissociation energy of individual SimCl is higher than that of SimI or SimBr, and the strength of HaB via the Cl atom in SimCl∙Y^−^ complexes is weaker than that in the complexes of SimI and SimBr with chloride. Yet, the dissociation of SimCl∙Y^−^ into Sim^−^ + ClY pairs requires less energy than that of SimX∙Cl^−^. This surprising result is attributed to the much more favorable energy changes in the reactions of Cl^+^ (formally released by SimCl) with X^−^ compared to the X^+^ + Cl^−^ reaction. Similar trends are likely observed in the reactions of *N*-halosuccinimides with other nucleophiles. This explains the higher reactivity of SimCl in solutions with various nucleophiles, its efficiency as a halogenating agent, and the difficulties in obtaining halogen-bonded complexes of this halogenated electrophile.

## 4. Materials and Methods

Commercially available *N*-chlorosuccinimide (SimCl), methyltriphenylphosphonium iodide (MePh_3_PI), n-tetrapropylammonium iodide (Pr_4_NI), methyltriphenylphosphonium bromide (MePh_3_PBr), hexane, and dichloromethane (all from TCI America), were used without additional purification. Vibrational spectra of were measured with a Perkin-Elmer Frontier FT-IR spectrometer with Universal ATR Sampling Accessory.

Single crystals **1**–**5** were prepared either by the evaporation of the solutions containing mixtures of reactants at room temperature in dichloromethane or by the diffusion of hexane into dichloromethane solution of the reactants at −73 °C. In particular, to prepare crystals **1**, 27 mg (0.2 mmol) SimCl was dissolved in 5 mL of dichloromethane and 71 mg (0.2 mmol) of MePh_3_PBr was dissolved (separately) in 2 mL of dichloromethane, and the solutions were combined. The slow evaporation of the mixture at room temperature resulted in the formation of crystals suitable for the single-crystals X-ray measurements. The X-ray structural analysis showed that they comprised HaB complexes of SimBr and chloride with MePh_3_P^+^ counter-ions. Single crystals **2**, comprising SimI·Cl^−^ complexes with Pr_4_N counter-ions, were prepared in a similar way by the evaporation of a mixture of 27 mg (0.2 mmol) SimCl and 63 mg (0.2 mmol) of Pr_4_NI. To prepare single crystals **3**, a dichloromethane solution (5 mL) containing 13 mg (0.1 mmol) of SimCl was cooled down to −78 °C in a Schlenk tube and combined at this temperature with a dichloromethane solution (2 mL) containing 40 mg (0.1 mmol) of MePh_3_PI. The combined solution was carefully layered with a 1:1 mixture of dichloromethane and hexane and then with hexane. The Schlenk tube was placed in the cool bath and kept at −73 °C. The diffusion of (layered) hexane into the dichloromethane solution of the mixture of SimCl and MePh_3_PI produced crystals **3**. A similar diffusion of hexane into a solution containing SimCl and MePh_3_PBr at −73 °C resulted in the formation of two types of crystals—(orthorhombic) prisms **4** and (triclinic) plates **5.** Both of them comprised HaB complexes in which chlorine and bromine were disordered between two positions (with somewhat different populations in **4** and **5**). The predominant form in both crystals (with populations of 0.82 and 0.94 in **4** and **5**, respectively) showed bromine atoms bonded to the nitrogen atom of succinimide. The minor fractions of bromine (0.18 and 0.06 in **4** and **5**, respectively) were located at the terminal position of the SimX·Y^−^ complex. The chlorine showed the opposite distributions between these two sites.

Single crystal structures for **1** and **2** were determined on a Bruker AXS D8 Quest diffractometer with a fixed chi angle, a sealed tube fine focus X-ray tube with Mo Kα radiation (*λ* = 0.71073 Å), a single crystal curved graphite incident beam monochromator, and a PhotonII area detector. Single crystal structures of **3**, **4**, and **5** were determined on a Bruker AXS D8 Quest 4 circle diffractometer, a microfocus X-ray tube with Cu Kα radiation (*λ* = 1.54178 Å), and a PhotonIII area detector. Both instruments were equipped with an Oxford Cryosystems low temperature device and examination and data collection of crystals were performed at 150 K. Reflections were indexed and processed, and the files were scaled and corrected for absorption using APEX5 [44]. The space groups were assigned using XPREP within the SHELXTL suite of programs, the structures were solved by dual methods using ShelXT and refined by full-matrix least-squares against *F*^2^ with all reflections using Shelxl2019 using the graphical interface Shelxle [45,46,47,48]. Crystals **1**–**3** were twinned or multi-component, see the SI for details. If not specified otherwise, H atoms attached to carbon and nitrogen atoms were positioned geometrically and constrained to ride on their parent atoms, with C-H bond distances of 1.00, 0.99, and 0.98 Å for aliphatic C-H, CH_2,_ and CH_3_ moieties, respectively. Methyl H atoms were allowed to rotate but not to tip to best fit the experimental electron density. U_iso_(H) values were set to a multiple of U_eq_(C) with 1.5 for CH_3_, and 1.2 for C-H units, respectively. Crystallographic, data collection and refinement details are listed in Table S1 in the SI.

Geometries of the complexes and their components were optimized without constraints via M06-2X/def2-tzvpp calculations (in dichloromethane, with a polarizable continuum model) using the Gaussian 09 suite of programs [49,50,51,52]. Our earlier studies [26,27] showed that such calculations provide good modeling of the HaB complexes, and the results are consistent with the experimental data. The absence of imaginary frequencies confirmed that the optimized structures represent true minima. The binding energies of the halide in the HaB complexes SimX·Y^−^ (i.e., the strength of the X⋯Y bond) were determined as: Δ*E* = E(SimX·Y^−^) − *E*(SimX) − *E*(Y^−^), where *E*(SimX·Y^−^), *E*(SIMX), and *E*(Y^−^) are energies, including zero-point energies, of the optimized complexes, SimX and halide, respectively. Since the formation of the complex is accompanied by distortion of the reactants, the binding energy represents a combination of preparation (distortion) energy, *E*_prep_, and interaction energy between distorted fragments, Δ*E*_int_. The energy scan in Figure 4 was performed using opt = modredundant option in Gaussian 09 starting from the coordinates of the optimized SimCl·I^−^ complex. In these calculations, the N-Cl distance was increased stepwise (starting from 1.685 Å) by 0.1 Å and the complexes were optimized at each N-Cl separation. Initially, the increase in the N-Cl separation was accompanied by the decrease in Cl-I separations from about 3.39 Å to 2.33 Å. During the first several steps, the decrease in Cl-I separations was so significant that the N-I distance also decreased to 4.683 Å (as compared to the N-I distance of 5.076 Å in the initial complex). Once Cl-I bond was fully developed, the N-I distances started to increase. A combination of the N-Cl bond dissociation and the Cl-I bond formation resulted in a small (about 0.3 kcal/mol) bump at steps 7–9. Then, similar calculations were repeated starting from the optimized geometry of the SimI·Cl^−^ complex. In this case, however, the I-Cl bonding in the starting complex was much stronger, so there was no decrease in the N-Cl distances and there was no bump on the energy scan.

Energies, as well as atomic coordinates of the calculated complexes are listed in the ESI. Atomic charges were calculated using the NBO method [38] implemented in the Gaussian 09 suite of programs. QTAIM analyses [39] were performed with Multiwfn [53] using wfn files generated by Gaussian 09. The results were visualized using the molecular graphics program VMD [54]. The rate constants of the halogen rearrangements were estimated via the Eyring (transition-state theory) equation as k = (kT/h)K^≠^, where k and h are the Boltzmann and Plank constants (transmission coefficient is assumed to be about 1), K^≠^ = exp(−ΔG^≠^/RT) [55], and the free energy of the separate Sim^−^ +XY pairs (which represent the highest-energy point on the reaction pathway, Figure S5 in the Supporting Information) relative to the free energy of SimX·Y^−^ was taken as the activation free (Gibbs) energy, ΔG^≠^.

## Figures and Tables

**Table 1:** Geometric characteristics of N-X (intramolecular) and X⋯Cl (intermolecular) bonds in crystals **1**–**5**.

Crystal	N-X⋯Cl Contact	d_N-X_, Å	d_X_⋯_Cl_, Å	∠N-X⋯Cl, deg
**1**	N-Br⋯Cl	1.919(5)	2.6593(17)	179.4(2)
**2**	N-I⋯Cl	2.141(3)	2.666(1)	178.99(8)
**3**	N-I⋯Cl	2.145(7)	2.681(2)	179.1(2)
**4**	N-Br[Cl]⋯Cl[Br] ^a^	1.935(3) [1.76(3)]	2.731(9) [2.75(3)]	177.4(2) [176.9(14)]
**5**	N-Br[Cl]⋯Cl[Br] ^a^	1.926(3) [1.89(5)]	2.687(4) [2.67(7)]	178.74(11) [166(2)]

^a^ Cl and Br atoms are disordered between two sites. E.s.d.’s are shown in parenthesis. Minor forms and their characteristics are shown in brackets.

**Table 2:** Selected characteristics of the optimized SimX·Y^−^ complexes.

SimX·Y^−^	d_N-X_, Å	d_X-Y_, Å	ΔE, kcal/mol ^a^	q_y,_ ^b^ *e*	q_x,_ ^c^ *e*
SimI·Cl^−^	2.159	2.659	−15.6	−0.72	0.33
SimBr·Cl^−^	1.884	2.793	−7.7	−0.86	0.27
SimCl·I^−^	1.685	3.401	−2.3	−0.95	0.22
SimCl·Br^−^	1.686	3.179	−2.9	−0.95	0.22
SimCl·Cl^−^	1.689	2.986	−3.6	−0.95	0.23

^a^ Binding energy of Y^−^ anion in the HaB complex, ΔE = E(SimX·Y^−^) − E(SimX) − E(Y^−^), where E(SimX·Y^−^), E(SIMX), and E(Y^−^) are energies, including zero-point energies, of the optimized complexes, SimX, and halide, respectively. ^b^ NBO charges on the terminal halogen (Y); ^c^ NBO charges on the middle halogen (X).

**Table 3:** Electron and energy densities, as well as ELF at the BCPs on the N-X and X⋯Y bond paths in the optimized SimX·Y^−^ complexes.

	N-X Bond			X⋯Y Bond		
SimX·Y^−^	*ρ*(r)	*H*(r)	ELF	*ρ*(r)	*H*(r)	ELF
SimI·Cl^−^	0.097	−0.038	0.385	0.052	−0.011	0.317
SimBr·Cl^−^	0.155	−0.100	0.612	0.033	−0.001	0.166
SimCl·I^−^	0.225	−0.180	0.809	0.013	0.001	0.062
SimCl·Br^−^	0.225	−0.181	0.807	0.015	0.001	0.062
SimCl·Cl^−^	0.224	−0.179	0.804	0.018	0.002	0.066

**Table 4:** Dissociation energy (in kcal/mol) of the N-X bond in the HaB complexes.

	SimX·Y^−^ → Sim^−^ +XY		SimX·Y^−^ → Sim^·^ + XY^·−^	
SimX·Y^−^	Δ*E*	Δ*G*	Δ*E*	Δ*G*
SimI·Cl^−^	31.2	21.4	64.5	52.9
SimBr·Cl^−^	26.7	16.6	58.0	46.1
SimCl·I^−^	15.8	7.4	49.1	38.2
SimCl·Br^−^	24.5	15.0	55.9	44.6
SimCl·Cl^−^	26.0	17.7	58.0	47.9

## Data Availability

Complete crystallographic data, in CIF format, have been deposited with the Cambridge Crystallographic Data Centre. CCDC 2415447-2415451 contains the supplementary crystallographic data for this paper. These data can be obtained free of charge via www.ccdc.cam.ac.uk/data_request/cif. All computational data are contained within the article or Supplementary Material.

## Supplementary Materials

The following supporting information can be downloaded at: https://www.mdpi.com/article/10.3390/molecules30030639/s1, Figure S1. FT-IR spectra of *N*-haloimides and their complexes. Table S1. Selected experimental and calculated vibrational frequencies in individual *N*-haloimides and their complexes; Table S2. Crystallographic, data collection, and refinement details [56,57]. Figures S2–S6. X-ray structures of **1**–**5**. Table S3. Bond lengths and dissociation energies. Table S4. Calculated free energy changes for X^+^ + Y^−^ reactions. Table S5. Energies, ZPE and (Gibbs) free energies of the optimized complexes and individual SimX molecules. Table S6. Atomic coordinates of the optimized complexes.
