# Supplementary material for: Halogen Bonding and Rearrangements in Complexes of N-Chlorosuccinimide with Halides
Source: Molecules. 2025 Jan 31;30(3):639. doi: 10.3390/molecules30030639 (PMC11819834; doi:10.3390/molecules30030639)
Supplement: Supplementary file 1 [file molecules-30-00639-s001.zip › molecules-3451401-supplementary.pdf]

Supporting Information
for
Halogen bonding and rearrangements in complexes of N-chlorosuccinimide with halides

Maison Hardin,[1] Matthias Zeller,[2] and Sergiy V. Rosokha[1*]

[1]*Department of Chemistry, Ball State University, Muncie, IN, 47306, USA*
[2]*Department of Chemistry, Purdue University West Lafayette, IN, 47907, USA*

* Correspondence: svrosokha@bsu.edu

Content

Figure S1. FT-IR spectra of individual N-haloimides and their complexes.

Table S1. Selected experimental and calculated vibrational frequencies in individual N-haloimides and their complexes.

Table 2. Crystallographic, data collection, and refinement details.

Figure S2. Crystal packing of **1.**

Figure S3. Crystal packing of **2**

Figure S4. X-ray structure and crystal packing of **3**.

Figure S5. X-ray structure and crystal packing of **4**.

Figure S6. X-ray structure and crystal packing of **5**.

Table S3. N-X Bond lengths and dissociation energies in the individual SimX molecules.

Table S4. Calculated free energy changes for $X^+ + Y^-$ reactions.

Table S5. Energies, zero-point-energies and (Gibbs) free energies of the optimized complexes and individual SimX molecules.

Table S6. Atomic coordinates of the optimized complexes

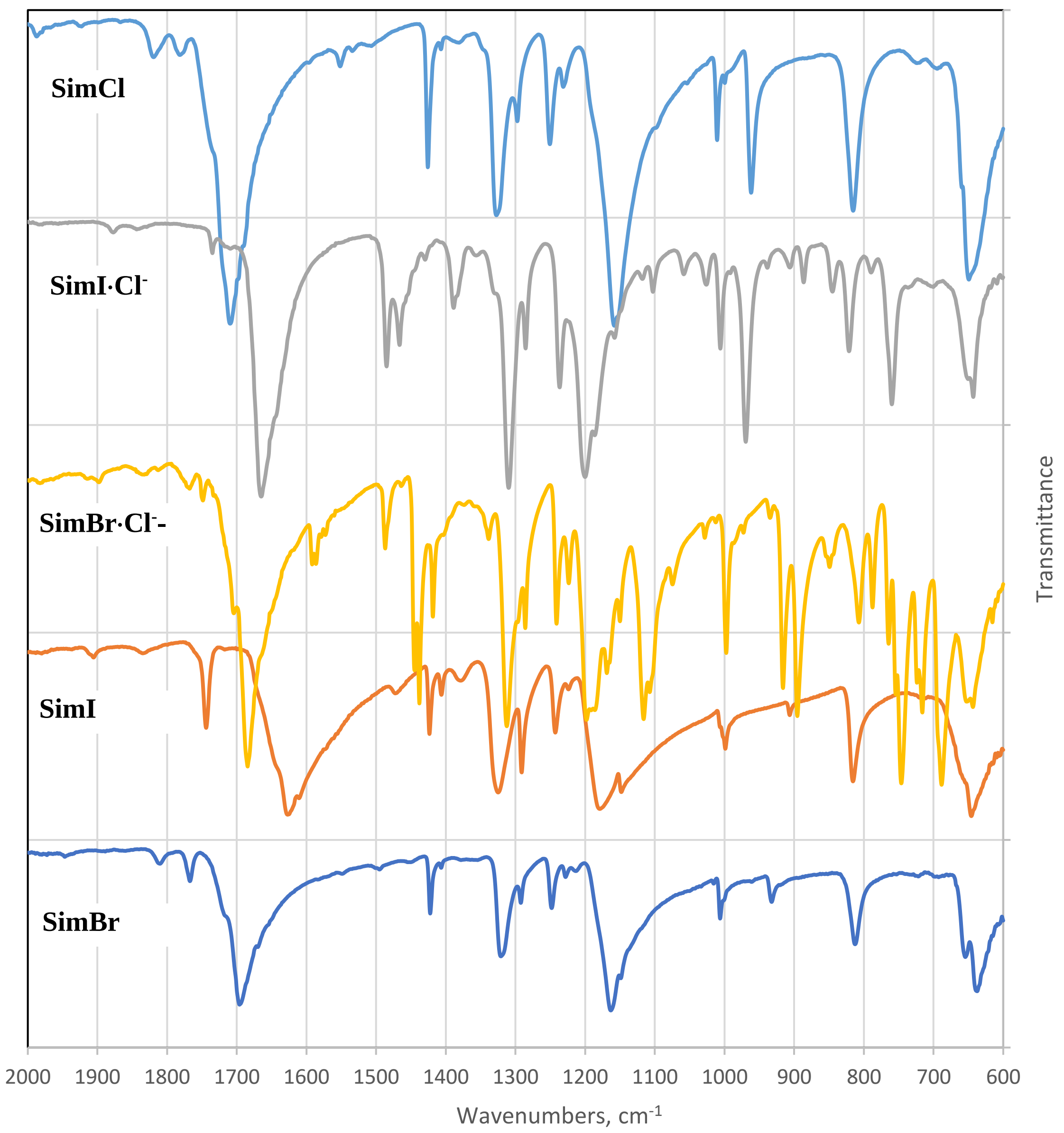

Figure S1. FT-IR spectra of N-halosuccinimides and their complexes

Table S1. Experimental and calculated CO and CN stretching frequencies[a] (in cm^{-1}) in individual N-halosuccinimides and their complexes with halides.

Vibration	SimCl	SimBr	SimI	SimCl·I$^-$	SimI·Cl$^-$	SimCl·Br$^-$	SimBr·Cl$^-$
$\nu_{CO\ (calc)}$*	1762	1757	1743	1750	1690	1749	1726
$\nu_{CO\ (exp)}$	1710	1696	1627	n/a	1665	n/a	1684
$\nu_{CN\ (calc)}$*	1160	1160	1165	1176	1252	1177	1197
$\nu_{CN\ (exp)}$	1158	1163	1179	n/a	1200	n/a	1198

*Main contribution to the normal modes.

Table S2. Crystallographic, data collection and refinement details.

	**1**	**2**	**3**	**4**	**5**
Chemical formula	$C_{19}H_{18}P\cdot C_4H_4BrNO_2\cdot Cl$	$C_{12}H_{28}N\cdot C_4H_4ClINO_2$	$C_{19}H_{18}P\cdot C_4H_4ClINO_2$	$C_{19}H_{18}P\cdot 0.178(C_4H_4ClNO_2)\cdot 0.822((C_4H_4BrNO_2)\cdot 0.178(Br)\cdot 0.822(Cl)$	$C_{19}H_{18}P\cdot 0.06(C_4H_4ClNO_2)\cdot 0.94((C_4H_4BrNO_2)\cdot 0.06(Br)\cdot 0.94(Cl)$
M_r	490.74	446.78	537.73	490.74	490.74
Crystal system, space group	Monoclinic, $P2_1$	Monoclinic, $P2_1/n$	Monoclinic, $P2_1$	Orthorhombic, $Pna2_1$	Monoclinic, $P2_1$
Temperature (K)	150	150	150	150	150
a, *b*, *c* (Å)	9.2329 (18), 13.703 (4), 9.475 (2)	12.3638 (6), 8.7758 (4), 19.5205 (9)	9.2254 (8), 14.0004 (10), 9.4972 (7)	17.5221 (8), 9.1611 (4), 13.7055 (6)	9.2390 (3), 13.7119 (4), 9.4712 (3)
(°)	115.522 (10)	105.525 (3)	114.601 (5)		115.4211 (8)
V (Å^3)	1081.8 (4)	2040.74 (17)	1115.30 (15)	2200.03 (17)	1083.68 (6)
Z	2	4	2	4	2
Radiation type	Mo $K\alpha$	Mo $K\alpha$	Cu $K\alpha$	Cu $K\alpha$	Cu $K\alpha$
(mm^{-1})	2.12	1.71	13.21	4.51	4.57
Crystal size (mm)	0.04 × 0.03 × 0.02	0.23 × 0.21 × 0.09	0.21 × 0.18 × 0.04	0.32 × 0.16 × 0.13	0.23 × 0.20 × 0.06
T_{min}, T_{max}	0.624, 0.746	0.639, 0.747	0.316, 0.754	0.504, 0.754	0.490, 0.754
No. of measured, independent and observed [$I > 2\sigma(I)$] reflections	18684, 6496, 4150	69418, 7798, 5575	26276, 4616, 4548	15864, 3970, 3891	14854, 4516, 4479
R_{int}	0.082	0.068	0.099	0.035	0.040
$(\sin \theta/\lambda)_{max}$ (Å^{-1})	0.714	0.770	0.639	0.639	0.639
$R[F^2 > 2\sigma(F^2)]$, $wR(F^2)$, S	0.055, 0.092, 0.99	0.049, 0.075, 1.13	0.044, 0.117, 1.10	0.025, 0.062, 1.05	0.024, 0.060, 1.03
No. of reflections	6496	7798	4616	3970	4516
No. of parameters	263	204	264	271	270

Data collection and refinement details:

Crystals **1**, **2** and **3**:

Crystals under investigation were found to be twinned or multi-component. The orientation matrices for the components were identified using the program Cell_Now.[1] The components were integrated using Saint and corrected for absorption using twinabs.

For crystal **1**, two components being related by an approximate 63.8 degree rotation around either the real or reciprocal b-axis. Integration and absorption correction resulting in the following statistics:

9016 data (1723 unique) involve domain 1 only, mean I/sigma 5.8

8972 data (1716 unique) involve domain 2 only, mean I/sigma 1.8

10117 data (2265 unique) involve 2 domains, mean I/sigma 3.8

The exact twin matrix identified by the integration program was found to be:

-1.00022 -0.00090 -0.00050

0.00191 -1.00000 -0.00016

0.88327 0.00033 1.00022

The structure was solved using dual methods with only the non-overlapping and corrrected reflections of component 1. The structure was refined using the hklf 4 routine with these reflections.[2]

For crystal **2** the two components being related by a 180° rotation around the real axis [2 0 7] or the reciprocal axis (-7 0 2). Integration and absorption correction resulting in the following statistics:

21629 data (2924 unique) involve domain 1 only, mean I/sigma 7.0

21559 data (2920 unique) involve domain 2 only, mean I/sigma 5.3

49551 data (7240 unique) involve 2 domains, mean I/sigma 5.8

13 data (13 unique) involve 3 domains, mean I/sigma 1.4

The exact twin matrix identified by the integration program was found to be:

0.56438 -0.00056 -0.43535

-0.00066 -1.00000 -0.00016

-1.56535 0.00196 -0.56438

The structure was solved using dual methods with only the non-overlapping reflections of component 1. The structure was refined using the hklf 5 routine with all reflections of component 1 (including the overlapping ones), resulting in a BASF value of 0.3840(1).[2]

For crystal **3** the two components being related by a 180 degree rotation around the reciprocal c-axis (0 0 1). Integration and absorption correction resulting in the following statistics:

16651 data (1760 unique) involve domain 1 only, mean I/sigma 26.1

16759 data (1763 unique) involve domain 2 only, mean I/sigma 19.1

9702 data (1197 unique) involve 2 domains, mean I/sigma 31.7

The exact twin matrix identified by the integration program was found to be:

-0.99988 -0.00013 0.00028

0.00062 -1.00000 0.00078

0.85733 0.00035 0.99988

The structure was solved using dual methods with only the non-overlapping reflections of component 1. The structure was refined using the hklf 5 routine with all reflections of component 1 (including the overlapping ones), resulting in a BASF value of 0.306(2) (Herbst-Irmer & Sheldrick, 1998).[2]

For all three structures, the R_{int} value given is for all reflections and is based on agreement

between observed single and composite intensities and those calculated from refined unique intensities and twin fractions (TWINABS (Sheldrick, 2012)).

Crystal **4** and **5**:

Chlorides and bromides are disordered with each other. ADPs were constrained to be identical for atoms in overlapping positions. Positions were freely refined.

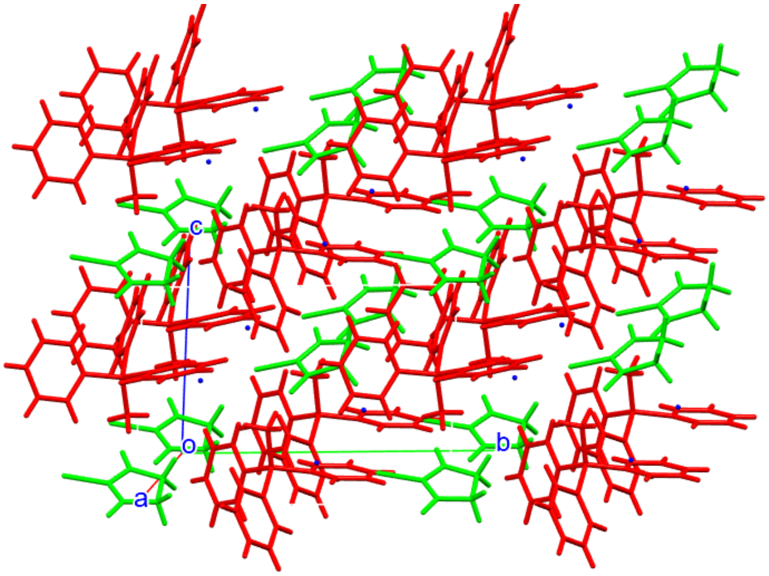

Figure S2. Crystal packing of **1.**

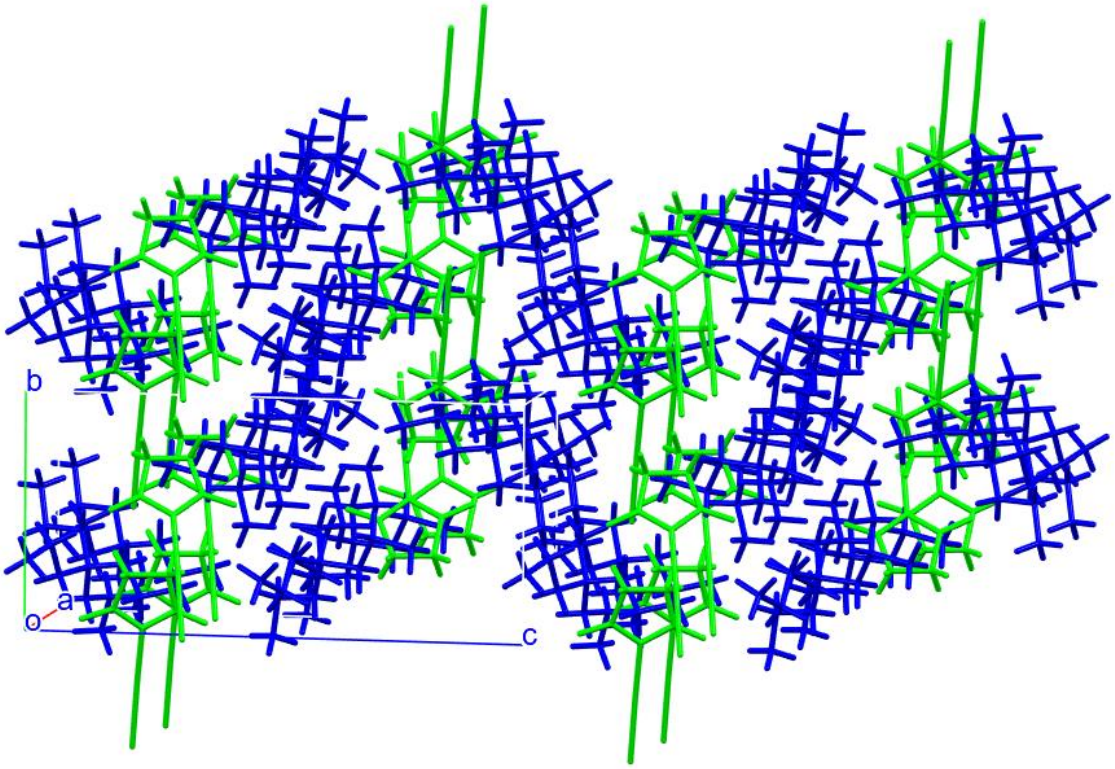

Figure S3. Crystal packing of **2.**

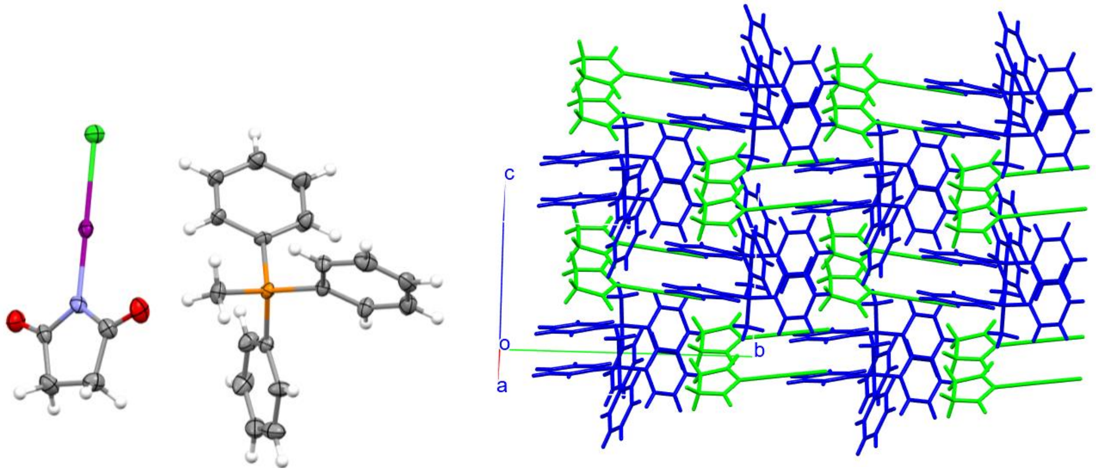

Figure S4. X-ray structure and crystal packing of **3**.

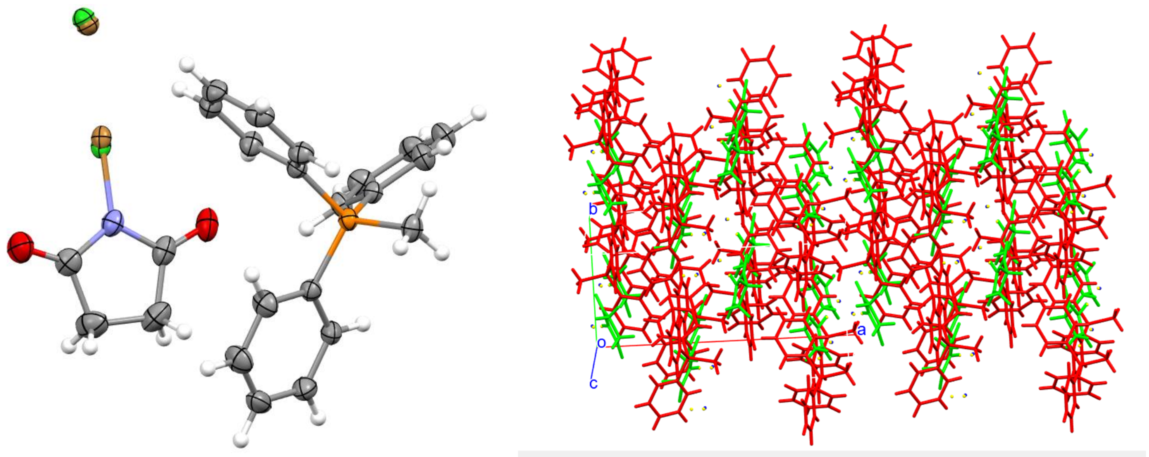

Figure S5. X-ray structure and crystal packing of **4**.

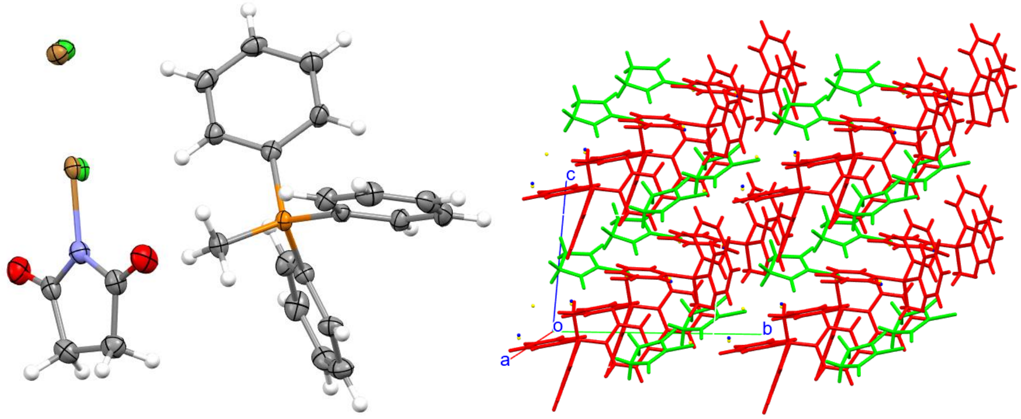

Figure S6. X-ray structure and crystal packing of **5**.

Table S3. N-X bond lengths (Å) and dissociation energies (in kcal/mol) of the optimized[a] SimX molecules.

		SimX → Sim^- + X^+		SimX → $Sim^·$ +$X^·$	
SimX	d_{N-X}	E	G	E	G
SimCl	1.670	214	206	78	69
SimBr	1.826	178	170	68	59
SimI	2.017	139	132	61	52

a) M06-2X/def2-tzvpp, PCM, CH_2Cl_2.

Table S4. Calculated free energy changes (in kcal/mol) for $X^+ + Y^- \rightarrow XY$ reactions.

$Cl^+ + I^- \rightarrow$ ClI	-196
$I^+ + Cl^- \rightarrow$ ClI	119
$Cl^+ + Br^- \rightarrow$ ClBr	186
$Br^+ + Cl^- \rightarrow$ ClBr	154

Table S5. Energies, zero-point-energies and (Gibbs) free energies of the optimized complexes and individual SimX molecules (a.u). (M06-2X/def-TZVPP computations, PCM, CH_2Cl_2)

	E	ZPE	G
SimI·Cl^-	-1118.07336	0.08182	-1118.03033
SimBr·Cl^-	-3394.61140	0.08251	-3394.56634
SimCl·I^-	-1118.04964	0.08261	-1118.00805
SimCl·Br^-	-3394.60851	0.08297	-3394.56387
SimCl·Cl^-	-1280.60231	0.08284	-1280.55794
SimI	-657.68789	0.08160	-657.64055
SimBr	-2934.23825	0.08205	-2934.18950
SimCl	-820.23583	0.08256	-820.18560
SIM^-	-360.19812	0.07942	-360.14861
Sim•	-359.97285	0.07706	-359.92710
Cl-Br	-3034.36877	0.00104	-3034.39135
Cl-I	-757.82411	0.00093	-757.84760
Cl-Cl	-920.36073	0.00131	-920.38120
$Cl-Br^-$•	-3034.54112	0.00047	-3034.56570
$Cl-I^-$•	-757.99340	0.00040	-758.01890
$Cl-Cl^-$•	-920.53191	0.00060	-920.55449
Cl^-	-460.36041	0	-460.37544
Br^-	-2574.36766	0	-2574.38384
I^-	-297.81015	0	-297.82699

Cl^{+}	-459.69356	0	-459.70859
Br^{+}	-2573.75369	0	-2573.76987
I^{+}	-297.26539	0	-297.28224

Table S4. Atomic coordinates of the optimized HaB complexes (M06-2X/def-TZVPP, PCM, CH_2Cl_2)

SimI·Cl^{-}

I	-0.96794100	0.00015200	-0.00014400
Cl	-3.62653200	-0.00012900	0.00028000
O	1.52372700	-2.27282500	-0.00002200
O	1.52462500	2.27288000	0.00001600
N	1.19149600	0.00009000	-0.00007800
C	1.95659000	-1.14291600	0.00007400
C	3.42890600	-0.76174900	0.00020000
H	3.89840300	-1.19982900	-0.87875700
H	3.89821200	-1.19972600	0.87930900
C	3.42921100	0.76102800	0.00010900
H	3.89881200	1.19892800	0.87909700
H	3.89878700	1.19878000	-0.87897000
C	1.95702800	1.14279200	0.00007700

SimBr·Cl^{-}

Br	-0.97135200	0.00021800	-0.00041000
O	1.22187600	-2.27460400	-0.10391500
O	1.22214900	2.27463300	0.10388100
N	0.91291800	0.00003000	-0.00003000
C	1.66914400	-1.15828700	-0.05501200
C	3.13254400	-0.76157800	-0.04578700
H	3.59189000	-1.13935800	-0.95738200
H	3.61651500	-1.24999800	0.79747000
C	3.13263400	0.76136000	0.04643700
H	3.59174700	1.13908000	0.95817300
H	3.61694400	1.24970500	-0.79667100
C	1.66928500	1.15825800	0.05516700
Cl	-3.76449300	0.00027200	-0.00063700

SimCl·I^{-}

O	-2.32682700	2.27813600	-0.02730500
O	-2.33042000	-2.27879600	-0.05863600
N	-2.04100200	-0.00038500	-0.06671500
C	-2.79001900	1.17216400	-0.02366500
C	-4.24606600	0.76497100	0.02920000
H	-4.75548800	1.20407500	-0.82611200
H	-4.68307700	1.18904000	0.93101800
C	-4.24728800	-0.76345700	0.01841500
H	-4.68498100	-1.19948300	0.91417300
H	-4.75743600	-1.18956800	-0.84301200
C	-2.79187900	-1.17224400	-0.04022900
I	3.03927900	-0.00226200	0.11734800
Cl	-0.35604900	-0.00129700	-0.08737700

SimCl·Br^{-}

O	-1.72751800	2.27403000	-0.14223500
O	-1.72678300	-2.27378700	0.14218300
N	-1.43762600	0.00016700	-0.00006200
C	-2.18897600	1.16902000	-0.07283500
C	-3.64600800	0.76248500	-0.04715500
H	-4.11987600	1.13862100	-0.95160600
H	-4.12032000	1.24735900	0.80366600
C	-3.64576200	-0.76284600	0.04739000
H	-4.11935700	-1.13913600	0.95191900
H	-4.12006500	-1.24786600	-0.80335300
C	-2.18860200	-1.16892400	0.07283200
Br	3.42760200	0.00054200	-0.00017600
Cl	0.24834400	0.00040700	-0.00013000

SimCl·Cl^{-}

Cl	-3.99816100	0.00000000	0.05319100
Cl	-1.01462000	-0.00018100	-0.07243200
O	0.96454700	-2.27808200	-0.01949300
O	0.96397800	2.27802500	-0.01942200
N	0.67369400	-0.00006600	-0.04603700
C	1.42445300	-1.16991700	-0.00620700
C	2.88063800	-0.76377500	0.05896200
C	2.88046400	0.76419900	0.05867400

C	1.42416800	1.16998000	-0.00613300	
H	3.39600300	-1.19704600	-0.79574000	
H	3.31168900	-1.19385400	0.96088000	
H	3.31180800	1.19476800	0.96021100	
H	3.39537100	1.19720300	-0.79644500	

References.

1. Sheldrick, G. M. CELL_NOW. Version 2008/4. University of Gottingen, Germany, **2008**.
2. Regine H.I; Sheldrick G.M. Refinement of Twinned Structures with SHELXL97. Acta Cryst. B54, **1998,** 443 - 449.
